# Role of aldehyde dehydrogenases, alcohol dehydrogenase 1B genotype, alcohol consumption, and their combination in breast cancer in East-Asian women

**DOI:** 10.1038/s41598-020-62361-9

**Published:** 2020-04-16

**Authors:** Boyoung Park, Ji-Hyun Kim, Eun Sook Lee, So-Youn Jung, See Youn Lee, Han-Sung Kang, Eun-Gyeong Lee, Jai Hong Han

**Affiliations:** 1 Research Institute, National Cancer Center, 323 Ilsan-ro, Ilsandong-gu, Goyang-si 10408 Korea; 2 Department of Medicine, Hanyang University College of Medicine, Seoul, Korea; 3 Hospital, National Cancer Center, 323 Ilsan-ro, Ilsandong-gu, Goyang-si, Gyeonggi-do 10408 Korea

**Keywords:** Lifestyle modification, Risk factors

## Abstract

The associations between genetic polymorphisms in *ADH1B* (rs1229984) and *ALDH2* (rs671), alcohol consumption, the effect of a combination of the two polymorphisms, and breast cancer risk were studied in a population of East-Asian women. In this study, 623 breast cancer cases and 1845 controls, aged 40 or above, were included. The association between *ALDH2* polymorphism and breast cancer risk was validated in 2143 breast cancer cases and 3977 controls. Alcohol consumption increased the risk of breast cancer regardless of *ADH1B* and *ALDH2* genotypes. The rs671 polymorphism of *ALDH2* was independently associated with increased breast cancer risk (OR = 1.27, 95% CI = 1.02–1.58 per increment of A). The *ADH1B* rs1229984 polymorphism, and combined effects of the rs671 and rs1229984 polymorphisms, did not reveal any significant association with breast cancer. Stratification by menopausal status revealed that rs671 gene polymorphisms were significantly associated with breast cancer only in postmenopausal women (OR = 1.45, 95% CI = 1.03–2.05 per increment of A). This is the first study to demonstrate an independent association between *ALDH2* gene variants and breast cancer in Asian women. Further studies are warranted to further elucidate the etiology of breast cancer as it relates to alcohol consumption in Asian women.

## Introduction

Alcohol consumption is a major risk factor that adversely affects the health of people worldwide^1^. In 2007, the International Agency for Research on Cancer (IARC) classified alcoholic beverages as group 1 carcinogens for the pharynx, larynx, esophagus, liver, colorectum, and female breast based on evidence on the toxicity of ethanol from epidemiological and animal studies^2^. Following alcohol intake, the enzyme alcohol dehydrogenase (*ADH*) oxidizes alcohol to acetaldehyde, which is highly toxic and carcinogenic to cells and tissues, and is detoxified to acetate by aldehyde dehydrogenases (*ALDH*)^3^. Thus, gene variants involved in alcohol metabolism and detoxification are also thought to affect the genetic susceptibility to alcohol-associated cancers. Among these, polymorphisms in the *ADH1B* and *ALDH2* genes are known to play a pivotal role in cancer development in combination with alcohol consumption^4^. The active *ADH1B* allele results in approximately 40 fold higher metabolism of alcohol to acetaldehyde compared to the inactive allele^5^. In cases with mutant *ALDH2* genes, *ALDH2* enzyme activity is suppressed and acetaldehyde metabolism in the liver is impaired; subsequent toxic reactions cause symptoms including facial flushing, nausea, headache, or palpitations after alcohol intake; those with deficient variants of *ALDH2* genes are likely to either consume less alcohol or be highly addicted^2,3,6,7^.

In general, epidemiological and animal studies have consistently correlated high alcohol intake with increased breast cancer risk in women^2^; however, the gene variants involved in alcohol metabolism or detoxification have only been inconclusively associated with breast cancer risk^8,9^. Considering increases in both alcohol consumption in women^10^ and incidence of breast cancer and related mortality in Asian countries^11^, in combination with the higher distribution of variant genes involved in alcohol metabolism or detoxification in the Asian population^12^, new studies exploring the associations and interactions among alcohol consumption, related ethanol-metabolizing genes, and breast cancer are warranted.

Thus, in the present study, we investigated the association among genetic, functionally established single nucleotide polymorphisms (SNPs) in *ADH1B* (rs1229984) and *ALDH2* (rs671), alcohol consumption, and their combined effects and breast cancer risk in an East-Asian population.

## Results

The general characteristics of the patients with breast cancer from the National Cancer Center (NCC) and age-matched controls from the Korean Genome Epidemiology Study (KoGES) are described in Table 1. Except age distribution and mean age, which were matched between cases and controls, the distribution of the other variables differed significantly between the cases and controls. In particular, the proportion of ever drinkers and those who drink >12 g ethanol/day was higher in the breast cancer group.

**Table 1:** Characteristics of the study population.

	Cases (*N* = 623)		Controls (*N* = 1845)		*P*-value
	*N*	%	*N*	%	
Age					0.995
40–49	373	59.9	1105	59.9	
50–59	195	31.3	575	31.2	
60-	55	8.8	165	8.9	
Mean age (SD)	49.14	(6.68)	49.12	(6.70)	0.959
Age at menarche					<0.001
≤15	523	83.9	896	48.6	
≥16	98	15.7	932	50.5	
Unknown	2	0.3	17	0.9	
Age at first full term pregnancy					<0.001
Nullipara	55	8.8	17	0.9	
≤23	62	10.0	575	31.2	
24–26	134	21.5	702	38.0	
≥27	367	58.9	528	28.6	
Unknown	5	0.8	23	1.2	
Age at menopause					<0.001
Premenopause	348	55.9	1085	58.8	
≤47	93	14.9	314	17.0	
48–50	55	8.8	253	13.7	
≥51	100	16.1	193	10.5	
Unknown	27	4.3	0	0.0	
Body mass index (kg/m^2^)					<0.001
≤23	306	49.1	520	28.2	
24–26.99	217	34.8	717	38.9	
≥27	88	14.1	608	33.0	
Unknown	12	1.9	0	0.0	
Smoking status					<0.001
Never	570	91.5	1756	95.2	
Ever	53	8.5	61	3.3	
Unknown	0	0.0	28	1.5	
Alcohol consumption					<0.001
Never	391	62.8	1252	67.9	
Ever	232	37.2	593	32.1	
≤12 g ethanol/day	150	24.1	467	25.3	
>12 g ethanol/day	43	6.9	56	3.0	
Unknown	39	6.3	70	3.8	
Pathology stage of breast cancer					
Stage 0	273	43.9			
Stage 1	201	32.2			
Stage 2	118	18.9			
Stage 3	22	3.5			
Unknown	9	1.5			
**Histology grade of breast cancer**					
Grade 1	67	10.7			
Grade 2	272	43.6			
Grade 3	161	25.9			
Unknown	123	19.7			
**Estrogen receptor status**					
Negative	269	43.1			
Positive	351	56.4			
Unknown	3	0.5			
**Progesterone receptor status**					
Negative	310	49.8			
Positive	310	49.7			
Unknown	3	0.5			

Table 2 presents the association between alcohol consumption, *ALDH2* rs671 polymorphism, *ADH1B* rs1229984 polymorphism, and breast cancer risk. Compared to the non-drinkers, the ever drinkers and those who consumed 12 g alcohol or more per day exhibited increased association with breast cancer (OR = 1.27, 95% CI = 1.01–1.61, *P*-value = 0.042; OR = 2.15, 95% CI = 1.28–3.59, *P*-value = 0.004). The rs671 genotype, although unassociated with breast cancer in an adjusted model, was significantly associated with breast cancer risk after the model was additionally adjusted for daily alcohol intake in patients with a GA or AA genotype compared with those presenting a GG homozygote (OR = 1.29, 95% CI = 1.00–1.65, *P*-value = 0.045) and per increment of A (OR = 1.27, 95% CI = 1.02–1.58, *P*-value = 0.032). The *ADH1B* rs1229984 polymorphism did not exhibit any significant association. The combined effects of the *ALDH2* rs671 and *ADH1B* rs1229984 polymorphisms are presented in Appendix Table 2. No significant association was observed between the combination of the two polymorphisms and breast cancer risk.

**Table 2:** Association among alcohol consumption, ALDH2 rs671 polymorphism, ADH1B rs1229984 polymorphism, and breast cancer risk.

	Cases		Controls		Odds ratio ^a^	*P*	Odds ratio ^b^	*P*	Odds ratio ^c^	*P*
	*N*	%	*N*	%						
**Alcohol consumption**										
Never	391	62.8	1252	67.9	1 (ref)		1 (ref)		1 (ref)	
Ever	232	37.2	593	32.1	1.25 (1.03–1.51)	0.020	1.27 (1.01–1.61)	0.042	1.38 (1.08–1.76)	0.010
≤12 g ethanol/day	150	24.1	467	25.3	1.03 (0.83–1.27)	0.799	1.11 (0.85–1.44)	0.441	1.20 (0.91–1.57)	0.189
>12 g ethanol/day	43	6.9	56	3.0	2.46 (1.62–3.71)	<0.001	2.15 (1.28–3.59)	0.004	2.38 (1.41–4.01)	0.001
**ALDH2 rs671 genotype**										
Codominant model										
GG	1318	71.4	428	68.7	1 (ref)		1 (ref)		1 (ref)	
GA	483	26.2	175	28.1	1.12 (0.91–1.37)	0.294	1.14 (0.89–1.45)	0.306	1.26 (0.98–1.63)	0.074
AA	44	2.4	20	3.2	1.40 (0.80–2.37)	0.222	1.44 (0.71–2.84)	0.297	1.67 (0.82–3.31)	0.151
Dominant model										
GG	1318	71.4	428	68.7	1 (ref)		1 (ref)		1 (ref)	
GA/AA	527	28.6	195	31.3	1.14 (0.96–1.35)	0.194	1.16 (0.91–1.47)	0.222	1.29 (1.00–1.65)	0.045
Additive model										
Increment of A	—	—	—	—	1.14 (0.93–1.39)	0.139	1.16 (0.94–1.42)	0.173	1.27 (1.02–1.58)	0.032
**ADH1B rs1229984 genotype**										
Co-dominant model										
TT	1036	56.2	358	57.8	1 (ref)		1 (ref)		1 (ref)	
TC	687	37.2	231	37.3	0.74 (0.80z–1.18)	0.780	0.91 (0.72–1.15)	0.446	0.91 (0.72–1.15)	0.435
CC	122	6.6	30	4.8	0.71 (0.46–1.07)	0.110	0.70 (0.42–1.14)	0.164	0.71 (0.42–1.16)	0.178
Dominant model										
TT	1036	56.2	358	57.8	1 (ref)		1 (ref)		1 (ref)	
TC/CC	809	43.8	261	47.2	0.93 (0.78–1.12)	0.495	0.88 (0.71–1.10)	0.275	0.88 (0.71–1.10)	0.274
Additive model										
Increment of C	—	—	—	—	0.91 (0.78–1.06)	0.224	0.88 (0.73–1.05)	0.165	0.88 (0.73–1.06)	0.171

^a^Unadjusted odds ratio; ^b^Adjusted for age at menarche, age at first full term pregnancy, age at menopause, body mass index, and smoking status; ^c^Additionally adjusted for alcohol consumption (for the association with ALDH2 or ADH1B) or ALDH2/ADH1B (for the association with alcohol consumption).

Table 3 lists the effect of alcohol consumption according to *ALDH2*/*ADH1B* polymorphism. In those homozygous for the GG allele of rs671, the effects of ever consuming alcohol or daily alcohol intake were associated with increased risk of breast cancer (OR = 1.54, 95% CI = 1.17–2.04, *P*-value = 0.002; OR = 2.57, 95% CI = 1.45–4.53, *P*-value = 0.001). In terms of the rs1229984 variant of the *ADH1B* gene, those homozygous for TT exhibited a significantly increased risk of breast cancer in both ever drinkers and those who daily consumed >12 g day alcohol (OR = 1.58, 95% CI = 1.15–2.17, *P*-value = 0.005; OR = 2.97, 95% CI = 1.49–5.94, *P*-value = 0.002); however, in women with a TC or CC genotype, no association was observed between alcohol consumption and breast cancer. When we combined the *ALDH2*/*ADH1B* polymorphisms, those homozygous for the major allele for both rs671 and rs1229984 (GG and TT) presented increased risk of breast cancer in ever drinkers (OR = 1.76, 95% CI = 1.22–2.57, *P*-value 0.003) and those who consumed 12 g alcohol or more per day (OR = 3.37, 95% CI = 1.59–7.22, *P*-value = 0.002).

**Table 3:** Association between alcohol consumption and breast cancer risk according to ALDH2 rs671 or ADH1B rs1229984 polymorphism.

	Cases		Controls		Odds ratio^a^	*P*-value	Odds ratio^b^	*P*-value
	*N*	%	*N*	%				
**ALDH2 rs671 genotype; GG**								
Alcohol consumption								
Never	225	52.6	801	60.8	1 (ref)		1 (ref)	
Ever	203	47.4	517	39.2	1.40 (1.12–1.74)	0.003	1.54 (1.17–2.04)	0.002
≤12 g ethanol/day	129	30.1	404	30.7	1.14 (0.89–1.45)	0.301	1.33 (0.98–1.81)	0.064
>12 g ethanol/day	41	9.6	52	3.9	2.81 (1.81–4.33)	<0.001	2.57 (1.45–4.53)	0.001
**ALDH2 rs671 genotype; GA/AA**								
Alcohol consumption								
Never	166	85.1	451	85.6	1 (ref)		1 (ref)	
Ever	29	14.9	76	14.4	1.04 (0.64–1.63)	0.879	1.34 (0.73–2.41)	0.336
≤12 g ethanol/day	21	10.8	63	12.0	0.91 (0.52–1.51)	0.711	1.15 (0.58–2.20)	0.685
>12 g ethanol/day	2	1.0	4	0.8	1.36 (0.19–7.03)	0.725	3.73 (0.45–24.11)	0.171
**ADH1B rs1229984 genotype; TT**								
Alcohol consumption								
Never	224	62.6	704	68.0	1 (ref)		1 (ref)	
Ever	134	37.4	332	32.0	1.27 (0.99–1.63)	0.063	1.58 (1.15–2.17)	0.005
≤12 g ethanol/day	85	23.7	264	25.5	1.01 (0.76–1.34)	0.936	1.32 (0.93–1.88)	0.121
>12 g ethanol/day	28	7.8	32	3.1	2.75 (1.61–4.67)	<0.001	2.97 (1.49–5.94)	0.002
**ADH1B rs1229984 genotype; TC/CC**								
Alcohol consumption								
Never	163	62.5	548	67.7	1 (ref)		1 (ref)	
Ever	98	37.5	261	32.3	0.26 (0.94–1.69)	0.116	1.24 (0.86–1.79)	0.250
≤12 g ethanol/day	65	24.9	203	25.1	1.08 (0.77–1.49)	0.661	1.10 (0.73–1.66)	0.634
>12 g ethanol/day	15	5.7	24	3.0	2.10 (1.06–4.06)	0.030	1.93 (0.81–4.48)	0.128
**ALDH2 rs671 & ADH1B rs1229984 genotype; GG and TT**								
Alcohol consumption								
Never	133	52.6	449	60.8	1 (ref)		1 (ref)	
Ever	120	47.4	289	39.2	1.40 (1.05–1.87)	0.021	1.76 (1.22–2.57)	0.003
≤12 g ethanol/day	75	29.6	226	30.6	1.12 (0.81–1.55)	0.493	1.46 (0.97–2.21)	0.068
>12 g ethanol/day	27	10.7	30	4.1	3.04 (1.74–5.30)	<0.001	3.37 (1.59–7.22)	0.002
**ALDH2 rs671 and ADH1B rs1229984 genotype; GA or TC**								
Alcohol consumption								
Never	216	67.9	682	72.3	1 (ref)		1 (ref)	
Ever	102	32.1	261	27.7	1.23 (0.93–1.62)	0.135	1.21 (0.85–1.70)	0.291
≤12 g ethanol/day	70	22	205	21.7	1.08 (0.79–1.47)	0.636	1.09 (0.74–1.60)	0.660
>12 g ethanol/day	14	4.4	23	2.4	1.92 (0.95–3.76)	0.060	1.76 (0.73–4.08)	0.197
**ALDH2 rs671 and ADH1B rs1229984 genotype; AA or CC**								
Alcohol consumption								
Never	39	79.6	121	73.8	1 (ref)		1 (ref)	
Ever	10	20.4	43	26.2	0.72 (0.32–1.52)	0.410	0.95 (0.32–2.68)	0.928
≤12 g ethanol/day	5	10.2	36	22	0.43 (0.14–1.09)	0.100	0.63 (0.14–2.25)	0.506
>12 g ethanol/day	2	4.1	3	1.8	2.07 (0.27–12.92)	0.435	1.70 (0.12–25.00)	0.681

^a^Unadjusted odds ratio; ^b^Adjusted for age, age at menarche, age at first full term pregnancy, age at menopause, body mass index, and smoking status.

Stratification by menopausal status revealed that alcohol consumption was significantly associated with breast cancer in premenopausal women (Table 4; OR = 1.75, 95% CI = 1.27–2.43, *P*-value = 0.001 for ever alcohol drinkers; OR = 1.53, 95% CI = 1.08–2.18, *P*-value = 0.018 for those consuming ≤12 g ethanol/day; OR = 2.87, 95% CI = 1.52–5.40, *P*-value = 0.001 for those consuming>12 g ethanol/day, compared with non-drinkers). Nevertheless, polymorphisms in the rs671 gene were significantly associated with breast cancer only in postmenopausal women with dominant alleles (OR = 1.63, 95% CI = 1.10–2.40, *P*-value = 0.014 for GA/AA genotype) and in the additive model (OR = 1.45, 95% CI = 1.03–2.05, *P*-value = 0.032 for one increment of A), but not for premenopausal women.

**Table 4:** Association among alcohol consumption, ALDH2 rs671 polymorphism, ADH1B rs1229984 polymorphism, and breast cancer risk based on menopausal status.

	Premenopausal						Postmenopausal					
	*N*	%	*N*	%	Odds ratio^a^	P	*N*	%	*N*	%	Odds ratio^a^	*P*
**Alcohol consumption**												
Never	197	56.6	695	64.1	1 (ref)		187	73.3	557	73.3	1 (ref)	
Ever	151	43.4	390	35.9	1.75 (1.27–2.43)	0.001	68	26.7	203	26.7	1.30 (0.85–1.96)	0.222
≤12 g ethanol/day	99	28.4	309	28.5	1.53 (1.08–2.18)	0.018	43	16.9	158	20.8	1.09 (0.69–1.73)	0.717
>12 g ethanol/day	33	9.5	41	3.8	2.87 (1.52–5.40)	0.001	9	3.5	15	2.0	2.40 (0.72–7.63)	0.144
**ALDH2 rs671 genotype**												
Co-dominant model												
GG	246	70.7	759	70	1 (ref)		168	65.9	559	73.6	1 (ref)	
GA	90	25.9	301	27.7	1.03 (0.73–1.46)	0.860	80	31.4	182	23.9	1.67 (1.12–2.49)	0.011
AA	12	3.4	25	2.3	2.12 (0.84–5.15)	0.102	7	2.7	19	2.5	1.12 (0.32–3.49)	0.846
Dominant model												
GG	246	70.7	759	70	1 (ref)		168	65.9	559	73.6	1 (ref)	
GA/AA	102	29.3	326	30	1.10 (0.78–1.54)	0.594	87	34.1	201	26.4	1.63 (1.10–2.40)	0.014
Additive model												
Increment of A	—	—	—	—	1.16 (0.86–1.56)	0.326	—	—	—	—	1.45 (1.03–2.05)	0.032
**ADH1B rs1229984 genotype**												
Co-dominant model												
TT	204	59.1	628	57.9	1 (ref)		142	55.9	408	53.7	1 (ref)	
TC	123	35.7	385	35.5	1.03 (0.75–1.41)	0.872	100	39.4	302	39.7	0.80 (0.56–1.15)	0.227
CC	18	5.2	72	6.6	0.85 (0.43–1.62)	0.637	12	4.7	50	6.6	0.56 (0.23–1.27)	0.182
Dominant model												
TT	204	59.1	628	57.9	1 (ref)		142	55.9	408	53.7	1 (ref)	
TC/CC	141	40.9	457	42.1	1.00 (0.74–1.35)	0.999	112	44.1	352	46.3	0.77 (0.54–1.09)	0.142
Additive model												
Increment of C	—	—	—	—	0.98 (0.76–1.25)	0.842	—	—	-	—	0.78 (0.58–1.04)	0.098
**ALDH2 rs671 and ADH1B rs1229984 genotype**												
GG and TT	148	42.9	438	40.4	1 (ref)		98	38.6	300	39.5	1 (ref)	
GA or TC	168	48.7	551	50.8	1.02 (0.74–1.40)	0.915	138	54.3	392	51.6	1.10 (0.77–1.59)	0.607
AA or CC	29	8.4	96	8.8	1.09 (0.61–1.90)	0.772	18	7.1	68	8.9	0.68 (0.32–1.40)	0.313

^a^Adjusted for age, age at menarche, age at first full term pregnancy, age at menopause, body mass index, smoking status, and alcohol consumption (for the association with ALDH2 or ADH1B) or ALDH2/ADH1B (for the association with alcohol consumption).

After investigating 2143 breast cancer cases and 3977 controls, the polymorphism in the *ALDH2* gene showed increased breast cancer risk in all genetic models (Appendix Table 3). The OR of GA/AA compared with that of GG was 1.13 (95% CI = 1.01–1.27, *P*-value = 0.034) and an increment in the A allele increased breast cancer risk by 1.14-fold (95% CI = 1.03–1.26, *P*-value = 0.013).

## Discussion

This is the first study suggesting that variants of the *ALDH2* gene (rs671) increase the risk of breast cancer independently, after adjusting for other covariates such as alcohol consumption, in Asian women, and in particular in postmenopausal women. In this study, both ever consuming alcohol and high dose alcohol consumption (>12 g/day) increased the risk of developing breast cancer. However, the association between alcohol consumption and breast cancer was more prominent in those with *ADH1B* His/His or those that were homozygous for the major allele in both *ADH1B* and *ALDH2*.

The association among ever consuming alcohol, high alcohol consumption (>12 g/day), and breast cancer in all women observed in the present study was in accordance with that in previous studies and reviews^8,13^. Previous studies have reported inconsistent results, wherein they evaluated whether menopausal status modifies the effect of the association between alcohol consumption and breast cancer risk^8,14^. In the present study, this association was observed only in premenopausal women. A possible explanation for the nonsignificant result in postmenopausal women could be that fewer women ever consumed alcohol or high alcohol content drinks; thus, the number was insufficient to statistically power our results. Considering that several studies suggest a modified effect of known genetic and environmental factors such as MPO gene polymorphism, family history, obesity, or alcohol use by menopausal status^14,15^, a detailed approach stratified by menopausal status with larger sample size is warranted.

*ADH1B* and *ALDH2* are the major enzymes involved in alcohol metabolism in humans. The role of the *ALDH2* gene has been widely studied in East-Asian countries because of the low prevalence of its variants in European populations. It has been reported that variants of the *ALDH2* gene are correlated with an increased susceptibility to numerous cancers, particularly of the upper digestive organs, such as esophageal or gastric cancer^16,17^; however, for other cancer types, the results were generally nonsignificant or inconsistent^16,18^. For breast cancer, only three studies have investigated the role of the *ALDH2* gene (rs671); however, none of them reported a significant association. In all three studies, the controls were recruited from women who visited the same hospital as the patients^19–21^. Although controls did not have cancer or other systemic diseases, and the proportion of alcohol consumption was in accordance with that in our study population, their inclusion may not substitute for the general population due to Berkson’s bias, a type of selection bias^22^. In our study population, the controls belonged to a population-based cohort, presumably free of Berkson’s bias, thus indicating a better reflection of females in general, and even after adjusting for alcohol consumption, polymorphisms in the *ALDH2* gene independently increased breast cancer risk. The association between *ALDH2* gene variants and breast cancer was validated in independent cases and all sampled control populations. In addition, compared with the previous studies in which the number of breast cancer patients and controls was less than 500^19–21^, the present study has a higher number of patients and 1:3 matching was used to increase the statistical power. In combination with the possible side effects of paclitaxel, a chemotherapeutic agent, due to alcohol’s role as a solvent^23^ women with the GA/AA genotype of *ALDH2* would be highly susceptible to not only breast cancer but also side effects of breast cancer treatment, and are therefore a population with a potentially high risk for delterious health effects.

*ADH1B* action results in higher blood acetaldehyde levels, which can increase DNA damage and lead to cancer^24^. In people with *ADH1B* His/His, the activity of *ADH1B* is enhanced, followed by rapid accumulation of acetaldehyde^25^. In epidemiological studies, *ADH1B* polymorphism has been associated with tumors of the upper digestive organs^4^ including esophageal cancer^26^. In terms of breast cancer, *ADH1B* itself did not present any association in most previous studies ^19,27,28^. One study reported a marginally protective association for *ADH1B* IVS1 + 896 A > G with breast cancer risk; however, the same SNP considered in this study did not exhibit any association^29^. Another study reported an interactive effect of *ADH1B* and alcohol consumption on breast cancer risk^27^. In the present study, although we did not identify a significant association between *ADH1B* polymorphism and breast cancer risk, a decreasing trend was observed in those with the C allele, in particular the homozygotes of the C allele, which was consistent with previous studies^29^. Another study reported that the effects of the *ADH1B* and *ALDH2* genes were mediated via alcohol consumption and decreased hepatocellular carcinoma risk^30^, which was not consistent with a previous meta-analysis^16^. In a study by Lie *et al*., the population included chronic hepatitis B carriers and the effect of alcohol was higher^30^ than seen in the present study or other studies conducted in the Korean population^31^. Considering the subgroup of women with possibly higher alcohol susceptibility, the effects of the *ADH1B* and *ALDH2* genes on alcohol drinking behavior and breast cancer risk might require further investigation.

In the present study, the association between alcohol consumption and breast cancer risk stratified by *ALDH2* and *ADH1B* polymorphism status, despite the presence of variants and alcohol intake, specifically a high dose of alcohol intake, increased the risk of breast cancer. Among the variants studied, those with a TT genotype for rs1229984 exhibited higher risk of breast cancer in ever drinkers or in those who consume more than 12 g of ethanol per day. These results are contradictory to those from a study in German women, wherein a reduction in breast cancer risk associated with 12 g or more alcohol consumption per day was observed^27^. The frequency of the His/Arg allele of *ADH1B* gene varies among populations, specifically the Asian and European populations^32^, and is based on diet or other lifestyle factors; moreover, other interacting genes may presumably lead to different results between studies.

It has been reported that both *ADH1B* and *ALDH2* present strong association with alcohol dependence. People with the A allele of rs671 display less alcohol dependence, possibly owing to the unpleasant effects of a higher flush response^33^, and those with the rs1229984 His allele also show a decreased risk of alcohol dependence, especially in Asian populations^32,34^. A genome-wide association study for alcohol consumption revealed that the rs671 A allele was associated with decreased prevalence of drinkers and number of drinks, and that the rs1229984 T allele was associated with fewer drinks but not with drinking status^35^. In this study population, women who carried the GG allele of rs671 consumed alcohol with >12 g ethanol/day more often compared to those with the GA/AA genotype (*P*-value < 0.001), as in previous studies^33,35^; however, their distribution was similar regardless of rs1229984 genotype. Previous studies included both men and women ^32,35^, and sex-differences in terms of alcohol intake pattern^35^ and alcohol metabolism may affect inconsistencies in the results.

Women who carried both the GG and TT genotypes for rs671 and rs1229984, respectively, presented a higher risk of breast cancer when they had ever consumed alcohol or more than 12 g ethanol per day, suggesting that for those with increased activity of the enzymes involved in alcohol metabolism and detoxification, the effects of alcohol consumption on breast cancer risk are increased. In these populations, alcohol would be eliminated faster than in other populations. Although highly active *ADH1B* and *ALDH2* may reduce the effects of alcohol and its toxic metabolites such as acetaldehyde, the effects of alcohol drinking such as intoxication and the recovery period may result in increased alcohol exposure.

Although a study conducted in Korea revealed marginally decreased association between the Lys allele of the *ALDH2* gene and breast cancer in postmenopausal women and no association in premenopausal women^20^, our results reveal an increased association between the Lys (A) allele of rs671 and breast cancer in postmenopausal women. The previous study included few premenopausal women, only 226 cases and 209 controls and only 60 cases of postmenopausal women carrying the Lys allele^20^; this may explain the lack of association. Regarding the *ADH1B* gene, other studies have demonstrated no significant association with breast cancer in postmenopausal women^36^ and those aged 50^27^, and these observations were consistent with our results.

This study has certain limitations. First, the existence of selection bias should be considered. To increase comparability, cases and controls were individually matched by age. Although external validity may be limited, the prevalence of variants in *ALDH2* and *ADH1B* genes is comparable with that in the general population^37^. Second, recall-bias could affect the results for measurements of alcohol consumption and other covariates. Some patients were recruited while receiving treatment, which may also affect the results. Third, the effects of other genes related to alcohol metabolism, such as Cytochrome P450 2E1, were not considered. Fourth, although this study included a larger sample size compared with previous studies on the association between *ALDH2*, *ADH1B*, alcohol consumption, and breast cancer, the statistical power may still be limited for elucidating gene–gene and gene–alcohol consumption interactions, and stratified analysis. In addition, some of the non-significant results from our subgroup analysis could be attributed to the low power of this study. Fifth, some variables that may affect both alcohol intake and breast cancer such as diet and exercise, were not considered because of limited available information and may have been incompletely adjusted for.

This study is the first to reveal an independent association between variants of the *ALDH2* gene and breast cancer in Asian women with results validated from a large number of cases and controls. Alcohol consumption increased breast cancer risk, but for cases homozygous for the major alleles of *ADH1B* or/and *ALDH2* polymorphisms, the increment was more prominent, suggesting that this was a high-risk population for breast cancers associated with alcohol consumption. Further studies with a larger sample size could help confirm the association between alcohol intake and gene interaction and breast cancer risk.

## Methods

### Study population

In total, we selected 750 women recently diagnosed with either invasive or *in situ* breast cancer, as confirmed by histology, at the NCC in Korea between February 2013 and October 2016. Healthy controls were selected from the KoGES, a population-based cohort constructed by the National Research Institute of Health (NIH), and the Centers for Disease Control and Prevention of Korea. Full details of the KoGES are described elsewhere^38^. Female control participants with a history of cancer were excluded, and 3986 women were considered for the study. Among these, 3977 controls provided information on alcohol consumption. All participants were from Korea, and written informed consent was obtained before enrolment. This study was approved by the National Cancer Center Institutional Review Board (IRB No. NCC 2015-0177). All procedures were performed in accordance with the Declaration of Helsinki.

### Data collection

All cases and controls were asked to complete a set of interviewer-assisted questionnaires on demographics, medical history, reproductive factors, and lifestyle (including alcohol intake). The subjects were questioned about alcohol consumption, their present drinking status, as well as the total duration of alcohol consumption for both present drinkers and those with a history of alcohol intake. In addition, the type of alcoholic beverage, weekly intake frequency, amount of alcohol consumed on each occasion, and serving sizes were evaluated. The subjects were classified according to drinking status (never and ever). Among ever drinkers, subjects were further classified according to alcohol intake: ≤12 g pure alcohol/day and >12 g pure alcohol/day based on the amount of ethanol in a standard drink.

### Genotyping and final study population

Genomic DNA was extracted from peripheral blood leukocytes and genotyped using the Korea Biobank Array with 833,000 SNPs. The Korea Biobank Array is a chip optimized for genetic studies in the Korean population. Full details on Korea Biobank Array are described elsewhere^39,40^. Of the 750 breast cancer cases and 3977 controls, 5 cases were excluded owing to a low call rate of <97% (*n* = 1) and family relationships (*n* = 4); after quality control, 745 cases and 3977 controls were evaluated. SNPs were excluded for low quality based on SNPolisher analysis (*n* = 36,816), low call rate <95% in either the cases or controls (*n* = 371), deviation from Hardy–Weinberg equilibrium (*p*-value <1 × 10^−6^) in the controls (*n* = 713), and monomorphic characteristics in our population (*n* = 107,204). In total, 688,196 markers were evaluated for quality control.

The SNPs rs671 and rs1229984, in the *ALDH2* and *ADH1B* genes, respectively, were analyzed. The cases and controls were individually matched by age at a 1:3 ratio, and ultimately 623 breast cancer cases and 1845 controls aged 40 or above were included in the final analysis. Discrepancies between the evaluated cases and controls were 0% and 0% for rs671 and 0% and 0.6% for rs1229984, with a *P*-value of 0.922. These SNPs had minor allelic frequencies of 15.5% and 25.2% and met the Hardy–Weinberg equilibrium in the control samples.

### Validation of the association between ALDH2 polymorphism and breast cancer

Data from 2143 breast cancer patients who were recruited as part of the KoGES were evaluated according to their sex, age, and genotype to validate the association between *ALDH2* polymorphism and breast cancer. Demographic and lifestyle information, including on alcohol intake, was unavailable for these patients. Peripheral blood DNA was genotyped using an Affymetrix Genome-wide Human SNP Array 6.0 (Affymetrix Inc., Santa Clara, CA, USA). As *ALDH2* (rs671) was not included in the array, genotype imputation for rs671 was performed. The SHAPEIT (v2.r837) was used for prephasing, and imputation was performed using IMPUTE2 (2.3.2). Data from the 1000 Genome Project phase 3 East-Asian Ancestry sample (*n* = 504) were applied as a reference panel. The quality control process and imputation was conducted as follows: among all subjects, those with a sex mismatch (*n* = 3) were excluded. No subjects showed a low call rate or family relationships. SNPs were excluded in cases of low call rate <95% (*n* = 151,323), deviation from Hardy–Weinberg equilibrium (*p*-value <1 × 10^−6^) in the controls (*n* = 897), or monomorphic traits in the population (*n* = 1710). In total, 513,738 markers were subjected to quality control. After imputation, the quality score was R^2^ = 0.57. The age distribution of the 2143 breast cancer patients and 3977 controls is presented in Appendix Table 1.

### Statistical analysis

The relationship between alcohol consumption and breast cancer risk was assessed with a simple logistic regression and a multiple logistic regression adjusted for age at menarche, age at first full term pregnancy, age at menopause, body mass index, smoking status, and rs671/rs1229984 genotype. The relationship between rs671 genotype, rs1229984 genotype, and breast cancer risk was additionally assessed with a simple and a multiple logistic regression adjusted for age at menarche, age at first full term pregnancy, age at menopause, body mass index, smoking status, and alcohol consumption. To assess the association between each SNP and breast cancer, the codominant, dominant, and additive models were considered. In addition, the combined effects of rs671 and rs1229984 genotype on breast cancer were assessed.

The *ALDH2* and *ADH1B* genotypes and alcohol intake status were collectively studied to evaluate their combined effect on breast cancer risk. Stratified by *ALDH2*, *ADH1B*, and a combination of *ALDH2*/*ADH1B* genotypes, the association among alcohol consumption itself, daily alcohol intake, and breast cancer was evaluated with odds ratio (OR) and *P*-value. In addition, after stratification by menopausal status, the relationship among alcohol consumption, rs671, and/or rs1229984 genotypes and breast cancer risk was assessed.

To validate the association between *ALDH2* polymorphism and breast cancer risk, the OR and *P*-values were presented, and adjusted for age between 2143 breast cancer cases and 3977 controls (all of the controls considered for the study) from KoGES. These analyses were performed with PLINK v.1.07^41^ and R statistical software version 3.2.2 (R Foundation, Vienna, Austria).

### Ethical approval and informed consent

Informed consent was collected for all cases and controls. This study was approved by the National Cancer Center Institutional Review Board (IRB No. NCC 2015-0177).

## Supplementary information

Supplementary Table 1-3.

## Data Availability

Data is available from the authors by request.
