# Supplementary material for: Role of aldehyde dehydrogenases, alcohol dehydrogenase 1B genotype, alcohol consumption, and their combination in breast cancer in East-Asian women
Source: Sci Rep. 2020 Apr 16;10:6564. doi: 10.1038/s41598-020-62361-9 (PMC7162854; doi:10.1038/s41598-020-62361-9)
Supplement: Supplementary file 1 — Supplementary Table 1-3. [file 41598_2020_62361_MOESM1_ESM.pdf]

Title: Role of aldehyde dehydrogenases, alcohol dehydrogenase 1B genotype, alcohol consumption, and their combination in breast cancer in East-Asian women

Author names:

Boyoung Park[1,2] (hayejine@hanmail.net)

Ji-Hyun Kim[1] (blrusti@ncc.re.kr)

Eun Sook Lee[1,3] (eslee@ncc.re.kr)

So-Youn Jung[1,3] (goje1@ncc.re.kr)

See youn Lee[1,3] (seeyoun@ncc.re.kr)

Han-Sung Kang[1,3] (rorerr@ncc.re.kr)

Eun-Gyeong Lee[3] (12772@ncc.re.kr)

Jai Hong Han[3] (51642@ncc.re.kr)

Affiliations:

[1]Research Institute, National Cancer Center, 323 Ilsan-ro, Ilsandong-gu, Goyang-si, 10408, Korea

[2]Department of Medicine, Hanyang University College of Medicine, Seoul, Korea

[3]Hospital, National Cancer Center, 323 Ilsan-ro, Ilsandong-gu, Goyang-si Gyeonggi-do, 10408, Korea

Appendix Table 1. Age distribution of validation study population including 2,143 breast cancer patients and 3,977 controls

	Cases (N=2,143)		Controls (N=3,977)		P-value
	N	%	N	%	
Age					<0.001
<45	778	36.4	1069	26.9	
45-49	539	25.1	744	18.7	
50-54	347	21.3	485	12.2	
55-59	203	4.3	541	13.6	
60-64	158	7.3	601	15.1	
≥65	118	5.5	537	13.5	

Appendix Table 2. Effect of combination of ALDH2 rs671 polymorphism and ADH1B rs1229984 polymorphism on breast cancer risk

	ADH1B rs1229984 genotype			
	TT		TC + CC	
	Case N (%)<br>Odds ratio [a]	Control N (%)<br>P-value	Case N (%)<br>Odds ratio [a]	Control N (%)<br>P-value
ALDH2 rs671 genotype				
GG	253 (40.9)	738 (40)	173 (27.9)	580 (31.4)
	1 (ref)		0.86 (0.65-1.12)	0.263
GA	97 (15.7)	274 (14.9)	77 (12.4)	209 (11.3)
	1.29 (0.92-1.79)	0.136	1.06 (0.73-1.53)	0.739
AA	8 (1.3)	24 (1.3)	11 (1.8)	20 (1.1)
	0.76 (0.22-2.32)	0.646	2.25 (0.89-5.55)	0.080

[a] Adjusted for age at menarche, age at first full term pregnancy, age at menopause, body mass index, smoking status, and alcohol consumption

Appendix Table 3. Validation results of the association between ALDH2 rs671 polymorphism and breast cancer risk in 2,143 cases and 3,977 controls

	Cases		Controls		Odds ratio [a]	P	Odds ratio [b]	P
	N	%	N	%				
ALDH2 rs671 genotype								
Co-dominant model								
GG	1458	71.4	2811	68.7	1 (ref)		1 (ref)	
GA	617	26.2	1075	28.1	1.11 (0.98-1.24)	0.091	1.11 (0.98-1.25)	0.097
AA	68	2.4	91	3.2	1.44 (1.04-1.98)	0.026	1.45 (1.04-2.01)	0.027
Dominant model								
GG	2075	71.4	3886	68.7	1 (ref)		1 (ref)	
GA/AA	68	28.6	91	31.3	1.13 (1.02-1.27)	0.032	1.13 (1.01-1.27)	0.034
Recessive model								
GG/GA	1458	71.4	2811	68.7	1 (ref)		1 (ref)	
AA	685	28.6	1166	31.3	1.40 (1.01-1.92)	0.039	1.41 (1.01-1.95)	0.041
Additive model								
Increment of A	-	-	-	-	1.14 (1.03-1.26)	0.012	1.14 (1.03-1.26)	0.013

[a] Unadjusted odds ratio; [b] Adjusted for age
